# The expanded application of CAR-T cell therapy for the treatment of multiple non-tumoral diseases

**DOI:** 10.1093/procel/pwad061

**Published:** 2023-12-26

**Authors:** Zhuoqun Liu, Yuchen Xiao, Jianjun Lyu, Duohui Jing, Liu Liu, Yanbin Fu, Wenxin Niu, Lingjing Jin, Chao Zhang

**Affiliations:** Fundamental Research Center, Shanghai Yangzhi Rehabilitation Hospital (Shanghai Sunshine Rehabilitation Center), School of Medicine, Tongji University, Shanghai 201619, China; Fundamental Research Center, Shanghai Yangzhi Rehabilitation Hospital (Shanghai Sunshine Rehabilitation Center), School of Medicine, Tongji University, Shanghai 201619, China; Hubei Topgene Research Institute of Hubei Topgene Biotechnology Co., Ltd., East Lake High-Tech Development Zone, Wuhan 430205, China; Shanghai Institute of Hematology, State Key Laboratory of Medical Genomics, National Research Center for Translational Medicine at Shanghai, Ruijin Hospital Affiliated to Shanghai Jiao Tong University School of Medicine, Shanghai 200025, China; Shanghai Yuhui Pharmaceutical Technology (Group) Co., Ltd., and Shanghai Ruishen Technology Development Co., Ltd., Shanghai 201203, China; Shanghai Cancer Institute, Department of Biliary-Pancreatic Surgery, Renji Hospital Affiliated to Shanghai Jiao Tong University School of Medicine, Shanghai 200127, China; Fundamental Research Center, Shanghai Yangzhi Rehabilitation Hospital (Shanghai Sunshine Rehabilitation Center), School of Medicine, Tongji University, Shanghai 201619, China; Fundamental Research Center, Shanghai Yangzhi Rehabilitation Hospital (Shanghai Sunshine Rehabilitation Center), School of Medicine, Tongji University, Shanghai 201619, China; Fundamental Research Center, Shanghai Yangzhi Rehabilitation Hospital (Shanghai Sunshine Rehabilitation Center), School of Medicine, Tongji University, Shanghai 201619, China

As a powerful cell-based therapeutic approach, CAR-T therapy was originally designed for treating acquired immunodeficiency syndrome (AIDS) (Baker et al., 2023), but had been strikingly successful in curing hematologic malignancies and multiple solid tumors. Numerous evidence has expanded the medical application of CAR-T therapy for the treatment of many other human diseases beyond cancer. In this article, we discuss the principle of CAR-T and enumerate the current application and limitation in oncology. Finally, we provide a comprehensive perspective of current advance and future directions of CAR-T in treating multiple non-tumoral diseases.

The mechanism of CAR-T cell therapy consists of precise target recognition, binding, and elimination. Engineered CAR-T cells first recognize and bind to target cells expressing specific surface antigens and then secrete a series of cytotoxins to directly kill the pathogenic cells or modulate the immune microenvironment to alleviate symptoms and disease progression. More specifically, CAR-T cells bind to either hematological or solid tumoral cells via certain antibody–antigen interactions and release perforin and granzyme, causing damage to the plasma membrane and cell death. CAR-T cells also release cytokines, such as interferon-gamma (IFN-γ) and tumor necrosis factor-alpha (TNF-α), which subsequently activate the host immune system to inhibit tumor growth and metastasis. In addition, CAR-T cells synergize with other immune cells to attack tumor cells through cytotoxicity mechanism. For example, the cytokines released by CAR-T cells could attract and activate macrophages via cell surface ligands and lead to the phagocytosis of tumor cells. The cytokines and chemical signals released by CAR-T cells also attract dendritic cells into the tumor microenvironment and boost their capability to deliver antigens to other immune cells, thus enhancing the elimination efficacy (Davila et al., 2014; Fischer and Bhattarai, 2021; Larson and Maus, 2021). In certain non-tumoral diseases, for example, autoimmune diseases, engineered CAR-T cells are capable to directly targeting certain autoantigens on the surface and eliminating morbid immune cells, thereby alleviating the symptoms of such disease (Beheshti et al., 2022; Mackensen et al., 2022; Muller et al., 2023). As a validated clinical approach to effectively reduce pathogen load and promote immune clearance of the infection in viral infective patients, CAR-T cells are designed to clean up viruses by recognizing and killing infected types of cells (Qi et al., 2020). Notably, in the overactive immune patient, the immune response to antiviral therapy and the ability to fight against infection is enhanced by the secretion of inhibitory cytokines by CAR-T cells, such as IFN-γ and transforming growth factor beta (TGF-β), which ultimately reduce the inflammatory response resulted from autoimmune attacks.

Currently, six autologous CAR-T cellular therapies have been approved by FDA for the treatment of hematological malignancies. Among them, CAR-T cell products targeting CD19 include Yescarta, Kymriah, Breyanzi, and Tecartus (Watanabe et al., 2022). B-cell precursor-derived acute lymphoblastic leukemia (B-ALL) became the first medical indication of CD19 CAR-T therapy. Subsequently, the application was expanded to B-cell non-Hodgkin lymphoma (B-NHL), including diffuse large B-cell lymphoma (DLBCL) (Sermer and Brentjens, 2019) and mantle cell lymphoma (Jiang et al., 2022). Unlike CD19 CAR-T cells, B-cell maturation antigen (BCMA) targeting CAR-T cell products, Abecma and Carvykti, are explored for relapsed/refractory multiple myeloma (r/r MM) (Lu and Jiang, 2022). These therapies have displayed high remission rates and acceptable toxicity in completed clinical trials targeting different syndromes. However, Kymriah showed a lower remission rate (ORR 52%, CR 40%) (Maude et al., 2018) in DLBCL patients than Yescarta (ORR 83%, CR 58%), and Breyanzi (ORR 73%, CR 53%). Kymriah also exhibited more obvious side effects than Yescarta and Breyanzi, including cytokine release syndrome (CRS) and immune effector cell-associated neurotoxicity syndrome (ICANS), which might account for its sale decline. Carvykti (ORR 97.9%, CR 82.5%) performed better than Abecma (ORR 73%, CR 33%) (Lu and Jiang, 2022) for curing MM patients. Compared with a 100% ORR of CAR-T cells for MM treatment, traditional chemotherapy regimens could only reach 70%–90%.

In addition to a better efficacy, CAR-T cells also owned several clinical advantages as follows: (1) Highly individualized: CAR-T cells are capable to recognize and attack cancer cells with high specificity and selectivity, while chemotherapeutic drugs simultaneously kill normal somatic cells with more cytotoxic side effects; (2) Durable therapeutic effect: CAR-T cells can persist inside the body with a single intravenous administration and exhibit a long-term durable therapeutic effect on morbid cells. In contrast, chemotherapeutic drugs require termly oral or intravenous administrations due to the short half-life *in vivo*, further exacerbating their side effects; (3) Immune memory effect: Once activated, CAR-T cells can not only eliminate existing target cells but also persist a lasting immune memory effect. Therefore, CAR-T cells can re-recognize and attack the reemergence of cancer cells in the future, providing a long-term lasting effect with reduced risk of recurrence. However, safety issues still exist for CAR-T cell therapies. In a phase 1 clinical trial of CAR T cells (NCT03089203) utilizing dominant-negative TGF-β receptor armoring for the treatment of castration-resistant prostate cancer, one patient unfortunately passed away due to the development of grade 4 CRS and concurrent sepsis (Narayan et al., 2022). It was also reported that a B-ALL patient receiving Kymriah relapsed and died 9 months after achieving complete remission because CD19 CAR-T cells accidentally transduced into leukemic B-cell clones resistant to CAR-T therapy (Ruella et al., 2018). This evidence highlights the importance of understanding the mechanism of drug action and the demand to develop safer and more efficient CAR-T therapies to combat such resistance and side effects.

In addition to CD19, many other promising targets including CD20, CD22, and GPRC5D also achieved promising therapeutic results (Fig. 1A). CAR-T cells targeting dual antigens are termed bispecific CAR-T and may lead to a better remission rate. The most popular bispecific CAR-T CD19/CD22 and CD19/CD20 have shown better success in several clinical trials (NCT03233854, NCT03196830, ChiCTR1800015575, NCT03097770, NCT03019055), particularly by solving the issues of disease relapse caused by antigen loss (Qu et al., 2022). However, CAR-T cell therapy is always expensive due to the personalized approach, with a pricing range from $370,000 to $475,000. Yescarta has currently shown the highest sale performance, reaching $1.16 billion in 2022. Yescarta becomes the first CAR-T therapy with sales exceeding $1 billion, while the sales of other CAR-T products, except Kymriah, continue to increase steadily. Despite its success in hematologic malignancies, the development of CAR-T cell therapy is constrained by several factors. First, poor therapeutic effect was seen for many solid tumors due to immunosuppressive tumor microenvironment and antigenic heterogeneity. Second, homogenization of CAR-T targets may lead to the risk of antigen escape and drug resistance. Last, the cost really limits its accessibility for most patients and impedes further benefit due to economic reason or health insurance issue.

**Figure 1.**
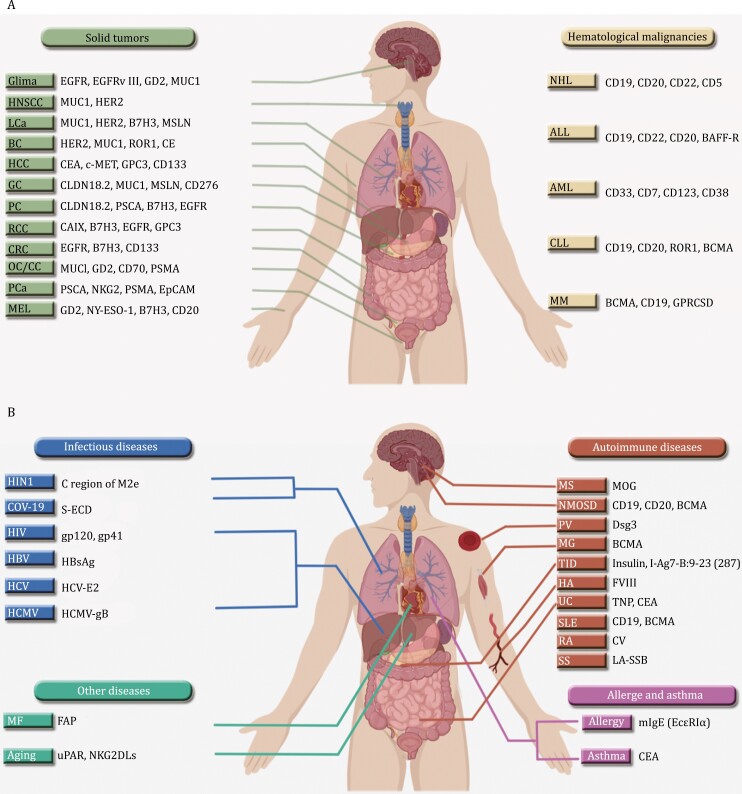
CAR-T therapies and drug targets for the treatment of various human diseases. (A) CAR-T therapies for the treatment of tumoral diseases. (B) CAR-T therapies for the treatment of non-tumoral diseases. Abbreviations: MF, myocardial fibrosis; HNSCC, head and neck squamous cell carcinoma; LCa, lung cancer; BC, breast cancer; HCC, hepatocellular carcinoma; PC, pancreatic cancer; GC, gastric cancer; RCC, renal cell carcinoma; CRC, colorectal cancer; OC, ovarian cancer; CC, cervical cancer; PCa, prostate cancer; MEL, melanoma; NHL, non-Hodgkin Lymphoma; ALL, acute lymphoblastic leukemia; AML, acute myelogenous leukemia; CLL, chronic lymphocytic leukemia; MM, multiple myeloma; MS, multiple sclerosis; NMOSD, neuromyelitis optica spectrum disorders; PV, polycythemia vera; MG, myasthenia gravis; T1D, type 1 diabetes; HA, hemophilia A; UC, ulcerative colitis; SLE, systemic lupus erythematosus; RA, rheumatoid arthritis; SS, Sjogren’s syndrome; ROR, receptor tyrosine kinase like orphan receptor; BCMA, B-cell maturation antigen; SLAMF7, signaling lymphocytic activation molecule F7; GPRC5D, G protein-coupled receptor class-C group-5 member-D; EGFR, epidermal growth factor receptor; GD2, ganglioside2; MUC1, mucin 1; HER2, human epidermal growth factor receptor 2; MSLN, mesothelin; CEA, carcinoembryonic antigen; CAIX, carbonic anhydrase IX; GPC3, glypican-3; CLDN, claudin; PSCA, prostate stem cell antigen; PSMA, prostate-specific membrane antigen; NY-ESO-1, New York esophageal squamous cell carcinoma-1; MOG, myelin oligodendrocyte glycoprotein; DSG3, Desmoglein 3; PV, pemphigus vulgaris; TNP, thymus nuclear protein; S-ECD, S protein Ectodomain; HBsAg, Hepatitis B surface antigen; HCMV, human cytomegalovirus. Photo was designed with Adobe Illustrator and modified by MedPeer.

As of November 2023, ClinicalTrials.gov has reported that 1,044 clinical trials of CAR-T cells are being globally conducted, with 573 in China, 327 in the USA, and 144 in other countries. Although the medical indication of CAR-T products for solid tumors has not yet been approved, many pharmaceutical companies have continuously begun to pursue this direction. In 2022, the number of approved clinical trials of CAR-T cells for solid tumors accounted for nearly half of the total, with a growth rate far exceeding that of hematological malignancies. Over 15 types of cancers, including glioma, breast cancer, pancreatic cancer, and tumors of the intestinal and digestive systems, are currently under clinical investigation. Representative antigens for CAR-T include Claudin 18.2, GPC3, HER2, EGFR, MSLN, and PSMA (Table 1). Overall, significant progression has been observed in multiple clinical trials of CAR-T cell therapy for solid tumors. Notably, CT041, the first CAR-T cell therapy targeting Claudin 18.2, has demonstrated a significant therapeutic effect on gastrointestinal tumors (NCT03874897) (Qi et al., 2022), with an ORR of 48.6% and a total disease control rate of 73.0%. The 6 months OS rate reaches 80.1%, and the median progression-free survival period is 3.7 months. Due to its excellent performance, CT041 now becomes the first CAR-T product in an approved phase II clinical trial. The Carl June et al. recently published phase I clinical trial of prostate-specific membrane antigen (PSMA) CAR-T therapy, in which the novel CAR-T overexpression with a dominant-negative effect on TGF-β RII (TGF-β RDN) offsets the immunosuppressive effect. Clinical studies have shown that 4 out of 13 patients showed a decrease of ≥30% in prostate-specific antigen levels. In addition, CT evaluation showed that five patients (38.5%) maintained disease stability during the 3-month imaging evaluation (NCT03089203) (Narayan et al., 2022). The therapeutic efficacy of other promising CAR-T cell therapies undergoing clinical studies is shown in Table S1. For solid tumors, the exploration of tumor-specific antigens and overcoming the immunosuppressive microenvironment become the most theoretical and technical challenges (Zhang et al., 2023). Scientists are developing more specific and efficient CAR-T cells with minimal off-target toxicity and investigating combinative approaches with other anti-cancer agents, such as immune checkpoint inhibitors or chemotherapy, to enhance the therapeutic efficacy and counteract the drug resistance.

**Table 1.** List of current and potential CAR-T therapies for treating non-tumoral human diseases.

NCT ID	Targeted antigen	Disease	Intervention	Phases, status
Validated targets				
NCT04146051	BCMA (CD269)	MG	Descartes-08 CAR-T	I&II, recruiting
NCT04561557		NMOSD	CT103A cell	I, recruiting
NCT05085431	BCMA/CD19	SS	CD19/BCMA CAR T	I, recruiting
NCT05085444		Scleroderma	CD19/BCMA CAR T	I, recruiting
NCT05085418		Immune nephritis	CD19/BCMA CAR T	I, recruiting
NCT05030779		SLE	CD19/BCMA CAR T	I, recruiting
NCT03030976	CD19	SLE	Anti-CD19 CAR-T	I, unknown
NCT03605238	CD19/CD20	Neuromyelitis optica spectrum disorder	Anti-CD19/anti-CD20 CAR-T	I, withdrawn
NCT04422912	Dsg3 surface immunoglobulin	Mucosal-dominant PV	DSG3-CAAR-T	I, recruiting
NCT03240328	CD4 binding site on gp-120	HIV	bNAbs (VRC01)-based CAR-T	I, recruiting
NCT04648046	CD4 binding site on gp-120		LVgp120duoCAR-T cells	I/IIa, recruiting
NCT03980691	CD4 binding site on gp-120	HIV	VC-CAR T cells combined with Chidamide	I, completed
NCT03617198	CD4 binding site on gp-120	HIV	CD4 CAR^+^CCR5 ZFN T-cells	I, active, not recruiting
NCT04863066	CD4 binding site on gp-120	HIV-1	Anti-gp120 105-scFV CAR-T cells	I, unknown status
NCT05784415	CD4 binding site on gp-120	HIV	CD4ζ-based modified T cells with and without extension Interleukin-2	I, active, not recruiting
NCT04324996	ACE2 & NKG2D	COVID-19	Bispecific anti-ACE2 and anti-NKG2D CAR-NK	Unknown status
Potential targets				
Targeted antigen	Disease	Targeting element	Co-stimulatory domain	References
I-Ag7-B:9–23 (R3) Complex	T1D	287A (scFv)-CAR-CD8^+^ T cells	CD28 and/or 4-1BB	(Zhang et al., 2019)
Insulin		Insulin-specific (scFv) CAR-Treg cells	CD28	(Tenspolde et al., 2019)
Dsg3	PV	Dsg3 CAAR-T cells	4-1BB	(Ellebrecht et al., 2016)
MOG	MS	Dsg3-targeted (scFv) CAAR-T cells	CD28	(Fransson et al., 2012)
CD19	SLE	CD19-targeted scFv CAR-T cells	CD28 or 4-1BB	(Mackensen et al., 2022)
CEA	UC	Anti-CEA (SCA431) scFv CAR-T cells	CD28	(Blat et al., 2014)
CD4 binding site on gp-120	HIV	Anti-CD4 CAR-T cells	CD28 or 4-1BB	(Zhen et al., 2017)
Env/gp120 glycans		Anti-CD4/CRD T cells	CD28	(Ghanem et al., 2018)
V1/V2 glycan loop		Anti-PGT145 CAR T cells	4-1BB	(Hale et al., 2017)
CD4-induced epitope on gp120/CD4 binding site		Anti-17b-scFv/Anti-17b CD4 CAR-T cells	CD28	(Liu et al., 2015)
CD4-induced epitope on gp120/CD4 binding site		Anti-mD1.22-G4S-m36.4 CAR-T cells	4-1BB	(Anthony-Gonda et al., 2019)
HBV S or L protein	HBV	αS-C8/αL-5a19 scFv cTCR T cell	CD28	(Bohne et al., 2008)
PreS1/LA14		anti-PreS1/LA14CART cells	CD28 and 41BB	(Guo et al., 2023)
HBV envelope protein on the surface of infected cells		19.79.6-scFv CAR T cells	CD28	(Kruse et al., 2018)
HCV/E2 glycoprotein	HCV	e137-scFv CAR T cells	CD28	(Sautto et al., 2016)
Viral fc receptors	HCMV	antiViral fc receptors scFv CAR T cell	CD28	(Proff et al., 2018)
HCMV glycoprotein B		anti-gB (SM5-1) scFv CAR T cells	4-1BB or CD28	(Olbrich et al., 2020)
EBNA-3C-derived peptide	EBV	TÜ165 scFv CAR T cells	CD28	(Dragon et al., 2020)
gp350		7A1-gp350CAR-T	CD28	(Slabik et al., 2020)
Receptor-binding domain of SARS-CoV-2 and pseudotyped SARS-CoV-2 S protein	SARS-CoV-2	CR3022 scFV CAR T cells	CD28, 4-1BB	(Zhu et al., 2021)
CEA	Allergic asthma	SCA431scFv-CAR-Tregs	CD28	(Quintarelli et al., 2018)
mIgE		FcεRIα and mIgE-targeted CAR T cells	4-1 BB and/or CD28	(Ward et al., 2018)
uPAR	Senescence-associated pathologies	Anti-uPAR scFv CAR-T	CD28	(Amor et al., 2020)
NKG2DLs		NKG2D CAR-T	4-1BB	(Yang et al., 2023)

Abbreviations: MG, myasthenia gravis; NMOSD, neuromyelitis optica spectrum disorder; SS, Sjogren’s syndrome; SLE, systemic lupus erythematosus; BCMA, B cell maturation antigen; LMP1, latent membrane protein 1; HIV, human immunodeficiency virus; UC, ulcerative colitis; PV, pemphigus vulgaris; UC, ulcerative colitis; HBV, hepatitis B virus; HCV, hepatitis C virus; HCMV, human cytomegalovirus; EBV, Epstein-Barr virus; CEA, carcinoembryonic antigen; MOG, myelin oligodendrocyte glycoprotein; Dsg3, Desmoglein 3.

The technical advance of gene editing and cell manufacturing are enabling the expansion of CAR-T immunotherapy beyond human cancer (Table 1). CAR-T cells have been explored as promising therapeutic approaches for autoimmune diseases, infectious diseases, allergic diseases, cardiac fibrosis, and aging-associated therapies (Fig. 1B). A variety of autoimmune diseases result from abnormal Treg cells. CAR-T cells can guide and stimulate Treg cells to the pathological site and ultimately suppress the syndrome-associated immune cells. This provides a highly effective and specific therapeutic option for severe autoimmune diseases, minimizing the side effects resulted from traditional treatment. Complete remission can also be achieved by direct elimination of aberrant autoantibody-producing plasma cells in systemic lupus erythematosus (SLE) (Kansal et al., 2019; Mackensen et al., 2022; Zhang et al., 2020). The New England Journal of Medicine reported the potential application of CAR-T therapy in SLE patients (Mougiakakos et al., 2021). Subsequently, the efficacy of CD19 CAR-T cell therapy in SLE was evaluated in five patients with severe or drug-resistant SLE and achieved long-term drug-free remission (Mackensen et al., 2022). In a most recent clinical study presented at the American Society of Hematology (ASH), researchers investigated the efficacy of CD19-targeted CAR-T therapy for the treatment of autoimmune diseases. The study included 15 refractory patients, consisting of 8 patients with SLE, 4 patients with systemic sclerosis (SSc), and 3 patients with idiopathic inflammatory myopathy (IIM). After a three-month treatment, all 8 SLE patients achieved CR, while all 3 IIM patients showed significant improvement and normalization of creatine kinase (CK) levels. Furthemore, the pathological condition in all 4 SSc patients decreased by 4.3 according to the criteria set by the European League Against Rheumatism (EULAR). More importantly, all 15 patients discontinued their usage of immunosuppressive drugs. Myasthenia gravis (MG), a chronic autoimmune neuromuscular disorder, is characterized by weakness and fatigue of arm or leg muscles, and problems with vision, mouth, and breathing. A recent study published in Lancet Neurology of July 2023 showed that the anti-BCMA CAR-T therapy was feasible in treating patients with MG. In December 2023, Haghikia et al. reported the first successful therapy of autologous anti-CD19 CAR-T in severe refractory, ACHR-positive systemic MG patients (Granit et al., 2023; Haghikia et al., 2023). Dual CAR-T of CD19 and CD20 (NCT03605238) or BCMA-targeted CAR-T (NCT04561557) are being investigated for the treatment of neuromyelitis optica spectrum disorders (NMOSD), a disease associated with optic nerve inflammation and myelopathy. CAR-T derivate therapies, such as CAR-Tregs and chimeric autoantibody receptor (CAAR-T) cells are also being evaluated for the treatment of autoimmune diseases. Unlike CAR-T cells that guide Tregs, CAR-Tregs are armed with CAR on Treg cell surface, and directly inhibit T-cell activation through the production of inhibitory cytokines upon recognition and binding to the corresponding antigen. In a mouse model of multiple sclerosis (MS), CAR-Tregs that target myelin oligodendrocyte glycoprotein attenuate the inflammatory response and effectively inhibit effector T-cells (Adabi et al., 2023). Moreover, the conventional treatment of Hemophilia A, an inherited bleeding coagulation disorder caused by deficiency of coagulation factor VIII is to control bleeding by supplementing coagulation factor VIII, which requires frequent supplementation and may lead to an excessive immune response and development of antibodies. Several studies have shown that CAR-Treg cells are capable of suppressing the production of autoantibodies and immune attack on clotting factor VIII (Yoon et al., 2017). In addition, CAR-Tregs could recognize specific antigens on the surface of insulin-producing β-cells within the pancreas to prevent the inflammatory response and abnormal immune attack resulting from autoimmune type 1 diabetes (T1D) (Radichev et al., 2020). It is noteworthy that CAR-Tregs have now only been investigated *in vitro* but not *in vivo*. Hence, an urgent need remains for clinical studies of CAR-Tregs for these human diseases.

As a promising cure for multiple autoimmune disorders, the major difference between CAAR and CAR is the antigenic recognition domain. CAAR replaces scFv of the extracellular region of CAR with recombinant autoantigens for binding autoantibodies or eliminating antigen-specific B cells. The Autoimmune Association has now documented over 150 autoimmune diseases and associated syndromes (from Autoimmune Association homepage) in which the patient’s immune system mistakenly attacks their own organs, leading to autoimmune flare-ups and severe tissue damage. CAARs can be designed to specifically recognize and bind to these aberrant targets and suppress the immune system by releasing granzymes and perforins that destroy cytotoxic T cells. For example, Pemphigus Vulgaris (PV), an autoimmune disorder characterized by the attack on the Dsg3 protein of the dermal and mucous membranes, leads to blister and ulcer in the skin. Ellebrecht et al introduced the binding domain of Dsg3 in CAAR-T cell surface and significantly attenuated the release of PV-associated autoantibodies and the degree of pathological syndrome (Ellebrecht et al., 2016). Preliminary clinical studies also confirmed the potential therapeutic efficacy of Dsg3-CAAR-T cell therapy for the treatment of PV (Adabi et al., 2023).

CAR-T cells are further engineered to treat infectious diseases by effectively eliminating invading pathogens. CD4-based CAR-T cells have achieved promising results against the Human Immunodeficiency Virus (HIV), but susceptibility to HIV infection limits their therapeutic efficacy and endogenous durability (Ma et al., 2020). Second-generation CD4^+^ CAR-T cells have been modified to control HIV infection by switching the extracellular region of the CAR to Nanobodies (Nabs). However, subsequent clinical results were unsatisfactory. Nonetheless, safe and sustained survival of CD4^+^ CAR-Ts was observed *in vitro* conditions (Ghanem et al., 2018; Leibman et al., 2017). Another clinical trial (NCT03980691) evaluated the safety and efficacy of concomitant therapy of Chiamide (a selective small molecule histone deacetylase inhibitor) with CAR-T cell therapy for HIV infection. Other trials evaluating various modified CD4-CAR-T cells have gradually initiated since 2017, such as NCT03240328, NCT03617198, and NCT04648046 (Seif et al., 2019). In addition, a Phase II study of universal ACE2 targeting CAR-NK cells for therapy of COVID-19-induced interstitial pneumonia is currently ongoing (NCT04324996). In the chronic hepatitis B virus-infected murine model, HBsAg-recognizing CAR-T cells effectively reduced HBs-DNA and HBsAg levels (Bertoletti and Tan, 2020). CAR-T cells that recognize HBV S, L, or envelope proteins can lyse HBV-replicating cells with minimal side effects. However, more preclinical studies in HBV-infected mice models are needed before launching the clinical investigation of CAR-T-cell therapy. Accordingly, CAR-T cells targeting the E2 glycoprotein of hepatitis C virus (HCV/E2) for the treatment of HCV-infected hepatocytes will be investigated in future studies. Moreover, CAR-T cells targeting the glycoprotein B (gB) of human cytomegalovirus (HCMV) have achieved promising results (Olbrich et al., 2020). Collectively, these results fully confirmed the strong potential of CAR-T cells in treating multiple chronic viral infections in human patients.

CAR-T therapy can also be employed to treat allergic asthma by targeting IgE-producing B cells. CD8^+^ CAR-T cells derived from FcεRIα showed low affinity for mediating primary T cell response against mIgE^+^ cells (Ward et al., 2018). CAR-Tregs specifically recognize antigens expressed in the airways of lungs and trigger immune resistance and prevent asthma-associated syndromes by aggregating and mobilizing in target tissues. Specifically, CAR-T cells could redirect Tregs to the lungs and attenuate asthma symptoms by reducing the expression of allergen-specific IgE and Th2 cytokines. It is reported that IL-13 receptor played an important role in pathogenesis and might be a valuable CAR-T target to treat allergic asthma (Adabi et al., 2023).

Furthermore, a recent study published in *Nature* reported the discovery of an endogenous target of cardiac fibroblasts, the fibroblast activation protein (FAP), by analyzing the gene expression profile of cardiac fibroblasts obtained from healthy and diseased human hearts. Aghajanian et al. designed FAP-specific CAR-T (mFAP CAR-T) cells and two doses of mFAP CAR-T administration resulted in dramatic remission in cardiac fibrosis and functional recovery upon injury in a murine model (Aghajanian et al., 2019). Subsequently, a study published in *Science* developed a novel therapeutic approach to treat injury-induced cardiac fibrosis in which they generated transient anti-fibrotic CAR-T cells *in vivo* by administration of T cell targeting lipid nanoparticle packed with modified mRNAs (Rurik et al., 2022). These studies provide proof of principle evidence for the development of immunotherapeutic approach for the treatment of cardiovascular disorders.

In addition to being effective in cardiac fibrosis, CAR-T therapy also successfully purges senescent murine cells with hepatic fibrosis to reverse senescence-associated pathologies by targeting uPAR, a protein widely expressed in senescent cells (Amor et al., 2020). Most recently, Yang et al. found that senescent cells upregulate natural killer tissue 2 member D ligands (NKG2DLs) in various tissues in senescent mice and non-human primates, regardless of the presence of stimuli that induce cellular senescence. They developed CAR-T cells targeting human NKG2DLs and demonstrated the endogenous efficacy in removing naturally occurring senescent cells in non-human primates without observing any adverse effects, which shed lights on the future development of medial invention to treat aging and age-related diseases (Yang et al., 2023).

Over the past decade, CAR-T cell therapies have made tremendous advances in anti-neoplastic diseases, particularly in the struggle against hematologic malignancies. Ongoing advances in pathology and their underlying molecular mechanisms are driving the widespread clinical application of CAR-T technology in nonmalignant tumors with great potential, particularly in infectious and immune-mediated diseases. A vast array of molecules has been validated as therapeutic targets for various diseases, making immune cell therapy an engaging and promising candidate for medical intervention. Various CAR-T cell regimens have advanced to clinical trials, offering the opportunity to improve or even cure chronic and degenerative diseases. Even though the treatment of HIV with CAR-T cells has not been successful to date, a few promising neo-concepts have been presented and warrant further exploration in the future. Investigation into how CAR-T cells act on immune dysfunction triggered by HIV or autoimmune diseases also contributes to the insight of tumor immune evasion mechanisms at the molecular level (Mylvaganam et al., 2019). CAR-T cells have been sequentially engineered to fight against HBV, HCV, CMV, EBV, and Aspergillus but remain in the early pre-clinical phase with confined efficacy (Table 1). Recently, the capability of CAR-T in targeting cellular senescence and pathological fibrosis has shown new possibilities, both of which are linked profoundly to chronic inflammation and cancer. Despite unresolved challenges concerning CAR-T cell therapies, such as the difficulties and cost associated with manufacturing, this approach shows great potential with promising clinical results and a substantial technical foundation. Ongoing studies hold promise for many irreversible conditions that could be potential candidates for innovative CAR-T cell therapies and sets the stage for a coming breakthrough clinical implementation in the future.

## Supplementary information

Supplementary data is available at *Protein & Cell Journal* online at https://doi.org/10.1093/procel/pwad061.

pwad061_suppl_Supplementary_Tables_S1

## Data Availability

All the data generated from this article are all included in this manuscript.
