# Supplementary material for: The expanded application of CAR-T cell therapy for the treatment of multiple non-tumoral diseases
Source: Protein Cell. 2023 Dec 26;15(9):633–41. doi: 10.1093/procel/pwad061 (PMC11365555; doi:10.1093/procel/pwad061)
Supplement: pwad061_suppl_Supplementary_Tables_S1 [file pwad061_suppl_supplementary_tables_s1.docx]

**Table S1: Therapeutic efficacy of FDA-approved CAR-T drugs and clinical trials**

| CAR-T products | Target | Application | Response rate | Year of approval | References |
| --- | --- | --- | --- | --- | --- |
| tisagenlecleucel | CD19 | R/R B-ALL | ORR 81%, CR 60% | 2017 | (Maude et al., 2018) |
|  |  |  |  |  |  |
|  |  | R/R DLBCL | ORR 52%, CR 40%, PR 12% | 2018 | (Schuster et al., 2019) |
|  |  |  |  |  |  |
|  |  |  |  |  |  |
|  |  | R/R FL | ORR 86%, CR 69% | 2021 | (Fowler et al., 2022) |
|  |  |  |  |  |  |
| axicabtagene ciloleucel | CD19 | R/R DLBCL, transformed FL | ORR 82%, CR 54% | 2017 | (Locke et al., 2019) |
|  |  | PMBCL, and HGBCL |  |  |  |
| brexucabtagene autoleucel | CD19 | R/R MCL | ORR 85%, CR 59% | 2020 | (Wang et al., 2020) |
|  |  |  |  |  |  |
| lisocabtagene maraleucel | CD19 | R/R DLBCL, HGBCL, PMBCL, and FL grade 3B | ORR 73%, CR 53% | 2021 | (Abramson et al., 2020) |
| idecabtagene vicleucel | BCMA | R/R MM | ORR 73%, CR 33% | 2021 | (Berdeja et al., 2021) |
| ciltacabtagene autoleucel | BCMA | R/R MM | ORR97%, CR 67% | 2022 | (Munshi et al., 2021) |
| MCARH109 | GPRC5D | MM | ORR 58% | 2022 | (Mailankody et al., 2022) |
| OriCAR-017 | GPRC5D | MM | ORR 100%, CR 60%,  PR 40% | 2023 | (Zhang et al., 2023a) |
| CART19/20 | CD20/CD19 | R/R NHL | ORR 91%, CR 73% | 2023 | (Puliafito et al., 2023) |
| GPRC5D-CART | GPRC5D | MM | ORR 91%, CR (63%), PR(27%) | 2023 | (Xia et al., 2023) |
| CAR-GPC3 T | GPC-3 | HCC | 1-year OS, 42% | 2020 | (Shi et al., 2020) |
| Mesothelin-Targeted CAR-T cell | mesothelin | MPM | 1-year overall survival, 83% | 2021 | (Adusumilli et al., 2021) |
| CT041 | Claudin18.2 | GC | ORR 48.6% , DCR 73.0% , 6-month OS 81.2% | 2022 | (Qi et al., 2022) |
| GD2 CAR-T 01 | GD2 | Neuroblastoma | ORR 63%, CR 33%,  PR 30% | 2023 | (Del Bufalo et al., 2023) |
| ALLO-316 | CD70 | RCC | CD70+ tumors  ORR 22%, DCR 100% | 2023 | (Srour et al., 2023) |
| C-CAR031 | GPC-3 | HCC | PR 80% (unconfirmed) | 2023 | (Zhang et al., 2023b) |
| CLDN6 CAR-T | CLDN6 | OC, TC, LUAD, UC | ORR 33% (unconfirmed),  CR 67% | 2022 |  |

Abbreviations:

NHL: Non-Hodgkin Lymphoma

PMBCL: Primary Mediastinal B-Cell Lymphoma

HGBCL: High-Grade B-Cell Lymphoma

B-ALL: B-Cell Acute Lymphoblastic Leukemia

DLBCL: Diffuse Large B-Cell Lymphoma

FL: Follicular Lymphoma

MCL: Mantle Cell Lymphoma

MM: Multiple Myeloma

RCC: Renal Cell Carcinoma

HCC: Hepatocellular Carcinoma

OC: Ovarian Cancer

TC: Testicular Cancer

LUAD: Lung Adenocarcinoma

UC: Uterine Cancer

GC: Gastric Cancer

MPM: Malignant Pleural Mesothelioma

R/R: Relapsed/Refractory

Abramson, J.S., Palomba, M.L., Gordon, L.I., Lunning, M.A., Wang, M., Arnason, J., Mehta, A., Purev, E., Maloney, D.G., Andreadis, C., et al. (2020). Lisocabtagene maraleucel for patients with relapsed or refractory large B-cell lymphomas (TRANSCEND NHL 001): a multicentre seamless design study. Lancet *396*, 839-852.

Adusumilli, P.S., Zauderer, M.G., Riviere, I., Solomon, S.B., Rusch, V.W., O'Cearbhaill, R.E., Zhu, A., Cheema, W., Chintala, N.K., Halton, E., et al. (2021). A Phase I Trial of Regional Mesothelin-Targeted CAR T-cell Therapy in Patients with Malignant Pleural Disease, in Combination with the Anti-PD-1 Agent Pembrolizumab. Cancer Discov *11*, 2748-2763.

Berdeja, J.G., Madduri, D., Usmani, S.Z., Jakubowiak, A., Agha, M., Cohen, A.D., Stewart, A.K., Hari, P., Htut, M., Lesokhin, A., et al. (2021). Ciltacabtagene autoleucel, a B-cell maturation antigen-directed chimeric antigen receptor T-cell therapy in patients with relapsed or refractory multiple myeloma (CARTITUDE-1): a phase 1b/2 open-label study. Lancet *398*, 314-324.

Del Bufalo, F., De Angelis, B., Caruana, I., Del Baldo, G., De Ioris, M.A., Serra, A., Mastronuzzi, A., Cefalo, M.G., Pagliara, D., Amicucci, M., et al. (2023). GD2-CART01 for Relapsed or Refractory High-Risk Neuroblastoma. N Engl J Med *388*, 1284-1295.

Fowler, N.H., Dickinson, M., Dreyling, M., Martinez-Lopez, J., Kolstad, A., Butler, J., Ghosh, M., Popplewell, L., Chavez, J.C., Bachy, E., et al. (2022). Tisagenlecleucel in adult relapsed or refractory follicular lymphoma: the phase 2 ELARA trial. Nat Med *28*, 325-332.

Locke, F.L., Ghobadi, A., Jacobson, C.A., Miklos, D.B., Lekakis, L.J., Oluwole, O.O., Lin, Y., Braunschweig, I., Hill, B.T., Timmerman, J.M., et al. (2019). Long-term safety and activity of axicabtagene ciloleucel in refractory large B-cell lymphoma (ZUMA-1): a single-arm, multicentre, phase 1-2 trial. Lancet Oncol *20*, 31-42.

Mackensen, A., Haanen, J., Koenecke, C., Alsdorf, W., Wagner-Drouet, E., Borchmann, P., Heudobler, D., Ferstl, B., Klobuch, S., Bokemeyer, C., et al. (2023). CLDN6-specific CAR-T cells plus amplifying RNA vaccine in relapsed or refractory solid tumors: the phase 1 BNT211-01 trial. Nat Med *29*, 2844-2853.

Mailankody, S., Devlin, S.M., Landa, J., Nath, K., Diamonte, C., Carstens, E.J., Russo, D., Auclair, R., Fitzgerald, L., Cadzin, B., et al. (2022). GPRC5D-Targeted CAR T Cells for Myeloma. N Engl J Med *387*, 1196-1206.

Maude, S.L., Laetsch, T.W., Buechner, J., Rives, S., Boyer, M., Bittencourt, H., Bader, P., Verneris, M.R., Stefanski, H.E., Myers, G.D., et al. (2018). Tisagenlecleucel in Children and Young Adults with B-Cell Lymphoblastic Leukemia. N Engl J Med *378*, 439-448.

McDermott, M.S., Gong, K.W., O'Brien, N.A., Conklin, D., Hoffstrom, B., Lu, M., Zhang, J., Luo, T., Jia, W., Hong, J.J., et al. (2022). Abstract 342: Development and characterization of a novel anti-CLDN6 antibody drug conjugate for the treatment of CLDN6 positive cancers. Cancer Research *82*, 342-342.

Munshi, N.C., Anderson, L.D., Jr., Shah, N., Madduri, D., Berdeja, J., Lonial, S., Raje, N., Lin, Y., Siegel, D., Oriol, A., et al. (2021). Idecabtagene Vicleucel in Relapsed and Refractory Multiple Myeloma. N Engl J Med *384*, 705-716.

Puliafito, B.R., Walthers, C., Ji, B., Ghafouri, S.N., Naparstek, J., Trent, J., Chen, J.M., Roshandell, M., Harris, C., Khericha, M., et al. (2023). Abstract CT023: Phase 1 trial of CD19/CD20 bispecific chimeric antigen receptor-engineered naïve/memory T cells for relapsed or refractory non-Hodgkin lymphoma. Cancer Research *83*, CT023-CT023.

Qi, C., Gong, J., Li, J., Liu, D., Qin, Y., Ge, S., Zhang, M., Peng, Z., Zhou, J., Cao, Y., et al. (2022). Claudin18.2-specific CAR T cells in gastrointestinal cancers: phase 1 trial interim results. Nat Med *28*, 1189-1198.

Schuster, S.J., Bishop, M.R., Tam, C.S., Waller, E.K., Borchmann, P., McGuirk, J.P., Jager, U., Jaglowski, S., Andreadis, C., Westin, J.R., et al. (2019). Tisagenlecleucel in Adult Relapsed or Refractory Diffuse Large B-Cell Lymphoma. N Engl J Med *380*, 45-56.

Shi, D., Shi, Y., Kaseb, A.O., Qi, X., Zhang, Y., Chi, J., Lu, Q., Gao, H., Jiang, H., Wang, H., et al. (2020). Chimeric Antigen Receptor-Glypican-3 T-Cell Therapy for Advanced Hepatocellular Carcinoma: Results of Phase I Trials. Clin Cancer Res *26*, 3979-3989.

Srour, S., Kotecha, R., Curti, B., Chahoud, J., Drakaki, A., Tang, L., Goyal, L., Prashad, S., Szenes, V., Norwood, K., et al. (2023). Abstract CT011: A phase 1 multicenter study (TRAVERSE) evaluating the safety and efficacy of ALLO-316 following conditioning regimen in pts with advanced or metastatic clear cell renal cell carcinoma (ccRCC). Cancer Research *83*, CT011-CT011.

Wang, M., Munoz, J., Goy, A., Locke, F.L., Jacobson, C.A., Hill, B.T., Timmerman, J.M., Holmes, H., Jaglowski, S., Flinn, I.W., et al. (2020). KTE-X19 CAR T-Cell Therapy in Relapsed or Refractory Mantle-Cell Lymphoma. N Engl J Med *382*, 1331-1342.

Xia, J., Li, H., Yan, Z., Zhou, D., Wang, Y., Qi, Y., Cao, J., Li, D., Cheng, H., Sang, W., et al. (2023). Anti-G Protein-Coupled Receptor, Class C Group 5 Member D Chimeric Antigen Receptor T Cells in Patients With Relapsed or Refractory Multiple Myeloma: A Single-Arm, Phase Ⅱ Trial. J Clin Oncol *41*, 2583-2593.

Zhang, M., Wei, G., Zhou, L., Zhou, J., Chen, S., Zhang, W., Wang, D., Luo, X., Cui, J., Huang, S., et al. (2023a). GPRC5D CAR T cells (OriCAR-017) in patients with relapsed or refractory multiple myeloma (POLARIS): a first-in-human, single-centre, single-arm, phase 1 trial. Lancet Haematol *10*, e107-e116.

Zhang, Q., Fu, Q., Cao, W., Xu, X., Xia, A., Huang, J., Zou, A., Zhu, J., Wang, F., Hong, Y., et al. (2023b). Abstract CT097: First report of preliminary safety, efficacy, and pharmacokinetics of C-CAR031 (GPC3-specific TGFβRIIDN CAR-T) in patients with advanced HCC. Cancer Research *83*, CT097-CT097.
